# Intensive care of the very old – questioning the relationship between illness severity and the moral imperative to deliver life-saving care

**DOI:** 10.1186/s13010-025-00198-8

**Published:** 2025-10-15

**Authors:** Gabriele Leonie Schwarz

**Affiliations:** 1 Department of Surgical Services, Intensive Care Unit, Haukeland University Hospital, Bergen, Norway; 2 Present Address: Department of Clinical Medicine, University of Bergen, Bergen, Norway

**Keywords:** Aged, 80 and over, Critical care, Frailty, Death, Medicalization

## Abstract

**Background:**

Intensive care provision to very old patients is rapidly growing owing to demographic changes and increasing treatment intensity. However, intensive care carries only questionable benefit for the oldest patients, and many of them die after prolonged organ support. Departing from a clinical perspective, this study aims to explore the drivers for the expansion of critical care in advanced age, despite widespread awareness of its potential harms to patients, their families, healthcare professionals, and society.

**Methods:**

Theoretical study into the possible consequences of the medicalization of ageing and dying on intensive care provision for very old patients, applying Ian Hacking’s concepts of human, interactive and natural, indifferent kinds as a philosophical framework.

**Results:**

The physiological consequences of ageing are a risk factor for falling critically ill, and for dying from critical illness, while age itself is not regarded as a disease, despite having recently been classified as such. Understanding old age as a human, interactive kind explains the medicalization of ageing and dying as a self-perpetuating process. Defining the natural processes of ageing and dying as disease results in a morally strong call for clinical efforts to provide life-saving care to very old patients despite its questionable overall benefit. As a consequence, adhering to this narrow, medically defined relationship between illness severity and treatment intensity results in vastly increased decisional uncertainty in advanced age compared to younger patient populations in intensive care units.

**Conclusion:**

Delivering the right level of care to very old patients with critical illness requires a more comprehensive clinical approach with philosophical concepts and social theories complementing medical scientific knowledge.

*How does a man decide in what order to abandon his life?”*

*Cormac McCarthy*,* No Country for Old Men*.

## Introductory case

A fit, independent, 86-year-old man, widower, farmer, no known previous health care contact, no medications, was admitted to the hospital with an obstructing mass in his large bowel. The surgeon on call made the diagnosis and scheduled the patient for urgent surgery. Due to several days of vomiting before admission, he had developed severe derangement in blood electrolytes and hydration. Therefore, he was transferred to the intensive care unit for pre-operative optimization. I was the intensivist on call and met an old man who was conscious, oriented, and cooperative, and very grateful for symptom relief with regards to nausea, vomiting and abdominal pain. When the surgeon got back to the patient to obtain consent, the patient stated frankly that he would choose to forego surgery. The surgeon attempted to explain to the patient that there likely was an underlying malignancy and to emphasize the risks related to refraining from urgent surgery. At this point in the conversation, the patient picked up his slipper, threw it at the surgeon, and uttered: “Leave me alone, I am tied up with dying. I am not ill and I don’t need a doctor. I am only asking for a quiet space to die the death I am meant to die.” After a brief multidisciplinary discussion, the patient’s right to forego treatment was respected, and the medical professionals’ duty to save his life was set aside.

The main determinant of the ethical dilemma in this case appeared to be the patient’s age.

This case illustrates the distinction between two of the three concepts of human ailment - disease and illness - and the corresponding different perspectives on the same condition [1, 2]. From the medical point of view, there was clear evidence of disease, a diagnosis was made, for which surgical treatment exists, with high rates of at least short-term success. The treating clinician might have even felt an obligation to treat due to the urgent nature of the condition and the limited time the patient had to reflect on the decision. However, the patient did not experience his condition as an illness, but as his personal way of dying, and he claimed his fundamental right to a dignified closure. Unlike many very old patients with critical illness, he retained capacity. He was able to communicate his will, but he felt forced to use strong measures to receive health care, which was in keeping with his personal goals of therapy. The third perspective – the societal point of view – is not equally obvious, but represents the larger context for the ethical dilemma the stakeholders in this case experienced.

This essay aims to explore the consequences of an increasing societal trend to classify and treat ageing and dying in advanced age as a disease, with a focus on intensive care provision for very old patients.

## Background and framework

*“How is the space of possible and actual action determined not just by physical and social barriers and opportunities*,* but also by the ways in which we conceptualize and realize who we are and what we may be*,* in this here and now?” Ian Hacking [3; p.285]*.

The Canadian philosopher and Holberg prize laureate Ian Hacking (1936–2023) developed the concepts ‘making up people’, dynamic nominalism, and the looping effect with particular interest in mental illness, but they are of more general applicability. ‘Making up people’ describes that the possibilities for people are bounded by what is imaginable and determined by what is described. The naming, i.e. the classification of people, is a dynamic, repetitive way where the names and the named emerge in interaction with each other, a process he called dynamic nominalism. He argued that human ‘interactive’ kinds are different from natural ‘indifferent’ kinds in the way that they respond to being made up. The iterative process in which the description of a human kind changes the people under the classification, which in turn enables or warrants a re-classification, he called the looping effect [3–5]. 

About one in five patients in Western intensive care units (ICUs) is very old (in this context, above the age of 80), and the proportion of very old intensive care patients is growing faster than demographic changes indicate, owing to more aggressive treatments being offered to very old patients [6, 7] Notwithstanding, the benefit of ICU admission remains controversial for this large patient group [8, 9] with a high risk of overtreatment inflicting significant harms to both patients, families, and society [10]. When offering aggressive treatments with questionable benefit to very old patients, many healthcare professionals experience a feeling of being driven by underlying dynamics beyond their control [11]; and families and patients might do so, too [12]. This perception of self-perpetuating dynamics suggests that Ian Hacking’s work on interactive human kinds, making up people, dynamic nominalism and the looping effect might provide a suitable frame to think about how medicalization of ageing and dying emerges and affects very old people, the last chapter of their life and their way of dying.

## Is age a disease?

Defining disease remains a difficult task for scholars, policymakers, healthcare professionals, and the general public [13, 14]; one might even argue that it is unnecessary or misleading [15]. Still, in many cases, it is straightforward to achieve agreement on “X is a disease” or “Y is not a disease” regardless of the theoretical approach. It is in the grey zones between health and disease, where the lack of a universal definition becomes apparent. But most authors agree that a disease is a harmful or at least undesirable deviation from normal biological structure and/or function.

Age in itself is not necessarily harmful, and whether it is undesirable or not is mainly dependent on the cultural context in which ageing is experienced. In Western societies with a seemingly ubiquitous focus on bodily youth and perfection, the connotations of age are mostly negative, resulting in a request for remedies to postpone or even cure ageing. In some Eastern and African societies, old age might as well be viewed as the blessing of longevity, a state of privilege and freedom from the duties of younger ages, and as an accumulation of experiences rather than an accumulation of deficits.

In clinical practice, both perspectives on ageing may equally result in a desire for advanced treatment modalities in the event of acute critical illness, and low- and middle-income countries seem to be even more prone to overtreatment of very old patients, especially highly respected persons, in intensive care units [16]. But also in European countries, the treatment intensity offered to very old patients in intensive care units is inversely correlated to GDP per capita [17].

Whether ageing is a deviation from normal bodily function depends on the reference population. Ageing is a universal consequence of being alive, with its biological processes already starting in foetal life [18]. ‘Not living up to old age’ is usually caused by conditions unquestionably classified as disease, disability or trauma; hence, advanced age in itself is rather a feature of health than disease.

However, the progressive reduction of physiological reserves puts the very old population at increased risk of developing disease and disability, and at higher risk for persistently reduced function and other unfavourable outcomes in the event of acute disease or trauma. Geriatric syndromes, like frailty, occur most often among the very old, but are not restricted to older age. The increased health-related risks of an ageing population translate into increased healthcare consumption, but healthcare in turn also makes, albeit a likely minor, contribution to life expectancy. Therefore, acknowledging the correlation of high age and health care consumption, I argue that ageing should be categorized as a risk factor, but not as a disease per se.

## How did age become a disease anyway?

The reasoning above, that age in itself is not a disease, is shared by health care professionals, policy makers, and laypeople alike [19, 20]. For the 11th revision of the International Classification of Diseases (ICD), the World Health Organisation proposed to replace the ICD-10 code R54 “senility” with MG2A “old age”. This term was withdrawn after vivid protest from a broad array of stakeholders with various interests, and the extension code XT9T “ageing-related” was included [21].

The process of defining new diseases and setting treatment thresholds is heavily influenced by conflicting interests – both financial, intellectual and reputational [22–24]. The global anti-ageing and longevity markets were each valued in the range of $ 20–60 billion USD (the annual United Nations budget was $ 3.6 billion USD) in 2024, with forecasted growth rates above 6% per year, and many large pharmaceutical companies are their major players. Additionally, the main body of research on physical and mental ageing is situated in the field of medicine. Both are strong forces directing the concept of ageing into the realm of healthcare, diverting the flow of resources in the same way.

Geriatric medicine is a quite new field, with speciality training having been formalized in many countries less than 20 years ago [25]. It has undoubtedly benefited the very old patient population to receive more holistic medical attention. They are patients with complex conditions which often involve several organ functions and are highly interlinked with other determinants of their well-being. Therefore, they are at increased risk of receiving inadequate care in modern, highly subspecialised, single-organ focused, guideline-regulated health care systems [26]. But this is not a unique feature of the very old patient population. Other populations with complex conditions, e.g. patients with multitrauma or drug addiction, benefit from receiving professional holistic advocacy, too.

Frailty, a geriatric syndrome encompassing the processes of biological ageing, has now been categorized as a disease in the ICD-11 under MG2A, “ageing-associated decline in intrinsic capacity”. Hence, wariness of ageism has led to categorizing age by biology rather than chronology. Consequently, with the 11th revision of the ICD, ageing has also formally entered the medical sphere as a disease through the frailty-labelled backdoor, driven by potent financial, intellectual, and reputational interests. From my - the intensivist’s - point of view, also biological age conceptualized as frailty, represents a risk factor, but not a disease [27]. The “growing focus of medicine on identifying and modifying risk […] can have significant benefits for individuals and public health, but it also carries real risks, not least of all the medicalization of normal life” [28].

Social constructionists argue that the biological concept of a disease is socially negotiated. And hence, that “…if a condition is disease in the social sense, we know immediately that those persons deserve special treatment.” [29] This does not at least apply vice versa. Even so, there is broad societal agreement that older people deserve special treatment; the conclusion that, therefore, age should be classified as a disease and dealt with in the medical system is not justified. The very old may deserve special treatment due to their lifelong contributions to society, their experience and insights, or because of other cultural and social norms. In Ian Hacking’s words: “…we first need to confront the point of social construction analyses. Don’t ask for the meaning, ask what’s the point.” [5].

## Understanding very old age as an interactive, human kind – the medicalization of ageing

The classes Ian Hacking calls human kinds are always value-laden with an intrinsic call for help or cure in contrast to indifferent, natural kinds. “[C]lassifying people works on people, changes them, and can even change their past. The process does not stop there. The people of a kind themselves are changed. Hence ‘we’, the experts, are forced to rethink our classifications.” [4] The argument that age categories are interactive kinds has recently been made [30]. 

The very old advance to becoming the cutting-edge human kind of the 2020th decade, reinforced by the ageism debate in the wake of the COVID-19 pandemic [21]. According to Ian Hacking’s work, a cutting-edge human kind is characterized by professional societies of experts, who study their conditions, by regular conferences and by scientific journals to which the experts contribute and which in turn define who these experts are. Consequently, the question arises: do we fail to help the very old “because all our endeavours assume that we are dealing with a scientific kind? […] There is a proper tension here, because one thrust of research into human kinds is to biologize them. […] This thrust is one of the more powerful themata in scientific thought. Its very success made us swell with optimism. We have immense confidence in its potential.” [4] 

Biologizing the human experience of advanced age promotes its medicalization, while strong forces make us believe in medical measures to alleviate its hardships. Medicalization describes a process when human problems or experiences become defined and treated as medical problems, usually in terms of illnesses, diseases, or syndromes” [23, 31]. Directing more and more medical attention towards the hardships of ageing and consequently diverting resources from other sectors has, in turn, fed back on how advanced age is experienced by the very old themselves in their daily life, and how their social surroundings meet them. This again leads to “…one of the most troubling results of medicalization [is] that it encourages medical solutions while ignoring or downplaying the social context of complicated problems” [23, 32].

How much more health could be gained for the very old population from increasing the resources directed towards loneliness, towards the ‘handicap of being slow’ in a society functioning at high analogue and digital speed, and towards the stigmata related to frailty?

## Diagnosing frailty and the looping effect – the medicalization of dying

Frailty comprises the age-related decline in physical capacity, involving both musculoskeletal and solid organ function. The decline in solid organ function may not affect daily life, but becomes apparent when exposed to the stress of acute illness. This functional decline is a continuum towards death. Therefore, in the very frail, even minor illness or bagatelle trauma may result in a life-threatening downward spiral. If we direct our medical focus only at the sum of physical derangements and diagnoses in these cases, death becomes the ultimate multi-organ failure, which is in sharp contrast to how very old patients might address their state, as for example being ‘full of days’. The first is clearly a negative, value-laden term, while the latter carries positive connotations of satisfaction and peace.

Ian Hacking explains that “… biologizing human kinds does not thereby make them immune to looping effects […] because people of the kind behave differently and so are different. That is to say the kind changes, and so there is new causal knowledge to be gained and perhaps, old knowledge to be jettisoned. […] Groups of experts now collaborate and say together they are members of the ‘helping professionals’[…]” [4]. Being classified as frail alters the way very old people perceive their state of being, influences their choices for living, and affects how they are treated within their social context. Medical attention to ageing people may aggravate this looping effect by adding the negative consequences of health care, especially the side effects of polypharmacy, as well as immobility, anxiety, and confusion.

Iwan Illich, the Austrian priest, philosopher and social critic, presented his analysis named the ‘Medical Nemesis’ in 1974. What then could seem a sinister, dystopic rage, has become an alarming realistic prediction: “Health […] designates the ability to adapt to changing environments, to growing up and to ageing, to healing when damaged, to suffering and to the peaceful expectation of death […] Man’s consciously lived fragility, individuality, and relatedness make the experience of pain, of sickness, and of death an integral part of his life. The ability to cope with this trio in autonomy is fundamental to his health. […] The true miracle of modern medicine is diabolical. It consists of making not only individuals but whole populations survive on inhumanely low levels of personal health.” [33].

His contemporary, the English priest and moral ethicist, Gordon Dunstan, expressed similar concerns in milder words, albeit also strongly appealing to the British Society of Intensive Care in 1984: “The success of intensive care is not, therefore to be measured only by the statistics of survival, as though each death was a medical failure. It is to be measured by the quality of lives preserved or restored; and by the quality of the dying of those in whose interest it is to die; and by the quality of human relationships involved in each death” [34]. His words remained largely unheard for the following four decades of intensive care development and practice. Most studies in intensive care are still designed to prove mortality reduction as their primary outcome. Regardless of the high proportion of patients dying in intensive care units, the quality of care received by the dying and their families is poorly addressed both in research, in clinical practice, and in health care governance [35]. Very old patients are often excluded from clinical intensive care trials. If they are included, it is usually not accounted for that they might regard death as a more favourable outcome than survival to permanent dependency [36].

In critically ill, very old patients, most clinical efforts are directed towards correcting the deranged physiology of the frail organism, largely neglecting all other determinants of human well-being. Intensive care involvement during the last days of life of very old patients has reached enormous numbers exceeding 50% in some Western hospital populations [37], and many very old patients die in intensive care units after a prolonged period of invasive organ support [9] as if death was an “industrial accident” [38]. Even though in obituaries often described as ‘dying peacefully with their loved ones at his/her side’, the reality of dying under invasive organ support in an intensive care unit is a different one. In addition to inflicting harm on patients, their families, and society, the experience of providing ‘futile care’ is also a major cause of conflicts, moral stress, burnout and poor staff retention among intensive care professionals [11].

## Illness severity and the moral imperative to deliver lifesaving medical care

*“… care is increasingly directed by protocols that minimise uncertainties. Contemporary complexity research shows the lack of a linear relationship between cause and effect*,* but doctors and health care systems persist in purveying a simplistic rhetoric […]. How many patients [and doctors] really understand the numbers needed to treat they are caught up in?” Iona Heath* [39].

Hannah Wunsch, an American epidemiologist and intensivist, utilized Frank-Starling curves to describe the relationship between intensive care provision and its effect on society. She explored the possibility of societal harm with excessive intensive care provision conceptualized as ‘falling of the Frank-Starling curve’ analogous to the progressive reduction in cardiac function, when overfilling of the heart occurs [40]. Similar curves appear to be true for individual patients in intensive care as well, with the curves of older patients peaking at lower treatment intensities.

**Fig. 1:**
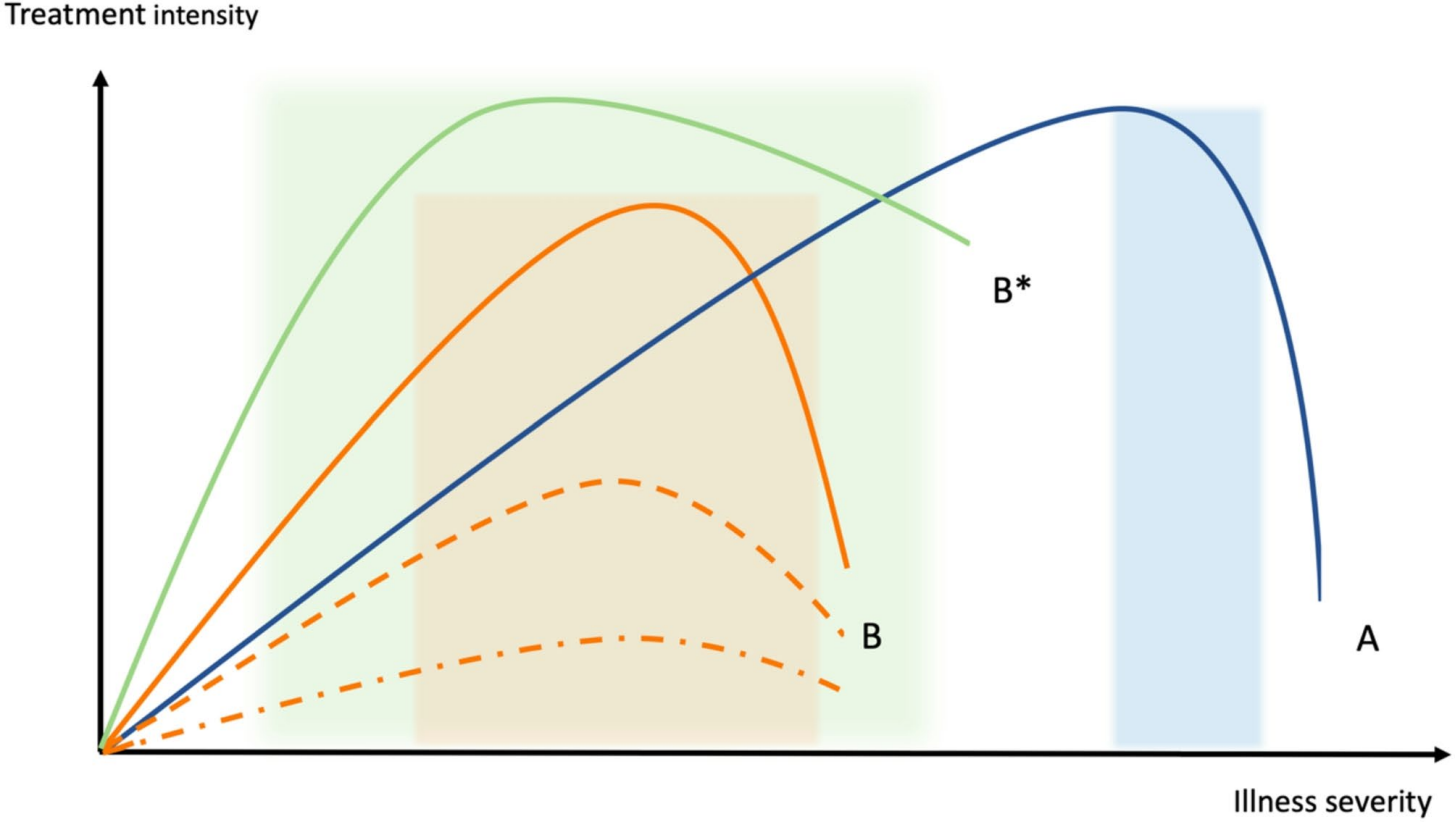
Schematic diagram of the relationship of illness severity and treatment intensity in ICU populations. The blue curve (**A**) is representative of a typical life-threatening condition in a young, previously healthy patient, peaking at very high illness severity. Older patients with acute critical illness are represented by a family of orange curves (**B**) owing to the considerable heterogeneity of this population. The green curve (B*) depicts the effect of treating age-related decline in organ function on par with acute organ dysfunction resulting in higher treatment intensity. Decisional uncertainty regarding treatment intensity is indicated by the corresponding shaded areas under the curves.

Ian Hacking observed that “[the] greater the moral connotations of a human kind, the greater potential for the looping effect” [4], which also seems to apply to frailty. There is a strong moral imperative associated with illness severity [41], calling for an obligation to save lives despite high personal and societal costs. I attempt to conceptualise the relationship between illness severity and treatment intensity using a similar family of curves (Fig. 1). At some point during treatment escalation, saturation will occur for every patient, beyond which excess treatment will add more harm than benefit. The blue curve (A) describes a young, fit patient with critical illness, where only at the extremes of physiological derangement and in the very likely absence of physiological effect of treatment, the clinician is released from the duty to save life, and organ support may be withheld or withdrawn. The shaded area represents the range of decisional uncertainty, which is typically narrow for young patients without underlying diseases, regardless of their critical condition. For very old patients, a whole family of orange curves (B) exist, depending on their underlying physical reserve and their personal goals of treatment. They may rise steeper than for patient A, since the frail organism requires organ support already at lower levels of illness severity, and they usually peak earlier, when the side effects of aggressive treatment outweigh the benefits. The shaded area of decisional uncertainty is much broader. Referring to the dynamics of the looping effect as discussed above, I argue that conceptualizing ageing as a disease pushes the curve of the very old patients upwards and to the left and additionally slows its decay (green curve B*); depicting that more aggressive treatments are provided to very old intensive care patients without new scientific evidence indicating increased benefit. This also adds largely to the range of related decisional uncertainty (Fig. 1).

Essentialising and simplification of the condition in which a patient presents is quite common in daily medical practice [42]. We might ask about the survival rate related to the diagnosis of the very old patient in the introductory case and then construct a conditional argument between diagnosis and the medically strongest indicated treatment option, while falling short of assessing the complexity of the larger context in which the patient presents to healthcare. The continuous decline in physical reserves and organ function not only puts the very old patient at increased risk in the event of critical illness. It may also be viewed and experienced as a continuous process of the ageing and dying organism, where the index diagnosis plays only a minor role.

## Concluding remarks

*“Concepts carry consequences – classifying things one way rather than another has important implications for the way we behave towards such things” L. Reznek*[43*; p.1]*.

Conceptualizing age as a disease carries numerous, both beneficial and harmful, consequences. I have argued that the harms may exceed the benefits for the very old patient in the event of acute critical illness warranting intensive care unit admission, especially when approaching the end of life. When such expansion of the concept of disease increases suffering, the harms inflicted transcend the patient in question, as we “deplete medicine and undermine the greatest asset in health care: trust.” [44].

The physiological derangement of acute illness can be classified as a natural kind subject to natural science. Still, the underlying drivers of intensive care provision need to be addressed by other methods. Hackings work provides one of several possible frames to explore and better understand the healthcare context and its dynamics. Delivering the right level of care to a critically ill, very old patient requires a broader understanding of the patient’s condition and the factors that drive medical decision-making.

The British general practitioner and writer Iona Heath summarizes the dynamics leading to increasing shortfall of modern health care: “The three trends of the industrialisation of health, the medicalisation of life, and the politicisation of medicine are intertwined and mutually reinforcing, and each depends on the pretence that we know much more than we do.” [39] To date in all areas of medicine, i.e. training, research, clinical practice, health care governance, as well as communication with patients and the public, the fact is not sufficiently appreciated, that medicine is not barely a natural science, but equally important subject to sources of knowledge brought forward by social and human sciences. Neglecting the need for a much more comprehensive approach leaves both patients and health care professionals with a vague feeling of being exposed to unexplained and uncontrolled underlying dynamics, resulting in an increasingly pressing experience of uncertainty and powerlessness when facing critical illness in advanced age.

## Data Availability

No datasets were generated or analysed during the current study.

## References

[1] Twaddle AC, Nordenfelt L. Disease, illness and sickness: three central concepts in the theory of health: a dialogue between Andrew twaddle and Lennart Nordenfelt. Linköping, Sweden: Linköping University, Dept. of Health and Society; 1994.

[2] Hofmann B. On the triad disease, illness and sickness. J Med Philos. 2002;27:651–73. 12607162 10.1076/jmep.27.6.651.13793

[3] Hacking I. Between Michel Foucault and Erving Goffman: between discourse in the abstract and face-to-face interaction. Econ Soc. 2004;33:277–302.

[4] Hacking I. The looping effects of human kinds. In: Sperber D, Premack D, Premack AJ, editors. Causal Cognition. Oxford University Press; 1996. p. 351–83. Available from: https://academic.oup.com/book/26284/chapter/194529638.

[5] Hacking I. The social construction of what? 7. print. Cambridge, Mass.: Harvard Univ. Press; 2001.

[6] Lilly CM, Swami S, Liu X, Riker RR, Badawi O. Five-year trends of critical care practice and outcomes. Chest. 2017;152:723–35. 28800866 10.1016/j.chest.2017.06.050

[7] Van Heerden PV, Sviri S, Beil M, Szczeklik W, De Lange D, Jung C, et al. The wave of very old people in the intensive care unit–a challenge in decision-making. J Crit Care. 2020;60:290–3. 32949896 10.1016/j.jcrc.2020.08.030

[8] Guidet B, Leblanc G, Simon T, Woimant M, Quenot JP, Ganansia O, et al. Effect of Systematic Intensive Care Unit Triage on Long-term Mortality Among Critically Ill Elderly Patients in France: A Randomized Clinical Trial. JAMA. 2017;318:1450–9. 28973065 10.1001/jama.2017.13889 PMC5710364

[9] Heyland D, Cook D, Bagshaw SM, Garland A, Stelfox HT, Mehta S, et al. The very elderly admitted to ICU: A quality finish? Crit Care Med. 2015;43:1352–60. 25901550 10.1097/CCM.0000000000001024

[10] Cardona-Morrell M, Kim J, Turner R, Anstey M, Mitchell I, Hillman K. Non-beneficial treatments in hospital at the end of life: a systematic review on extent of the problem. Int J Qual Health Care. 2016;28:456–69. 27353273 10.1093/intqhc/mzw060

[11] Boulton AJ, Slowther A-M, Yeung J, Bassford C. Moral distress among intensive care unit professions in the UK: a mixed-methods study. BMJ Open. 2023;13:e068918. 10.1136/bmjopen-2022-068918 PMC10151959 37185186

[12] Aronson L. Beyond code status. N Engl J Med. 2024;390:1451–3. 38647052 10.1056/NEJMp2314068

[13] Hofmann B. Complexity of the concept of disease as shown through rival theoretical frameworks. Theor Med Bioeth. 2001;22:211–36. 11499496 10.1023/a:1011416302494

[14] Sadegh-Zadeh K. Fuzzy health, illness, and disease. J Med Philos. 2000;25:605–38. 11035544 10.1076/0360-5310(200010)25:5;1-W;FT605

[15] Hesslow G. Do we need a concept of disease? Theoret Med. 1993;14:1–14. 8506536 10.1007/BF00993984

[16] Smith D. Nelson Mandela leaves hosptial in South Africa. The Guardian. 2013. Available from: https://www.theguardian.com/world/2013/sep/01/nelson-mandela-leaves-hospital-south-africa.

[17] Guidet B, Flaatten H, Boumendil A, Morandi A, Andersen FH, Artigas A, et al. Withholding or withdrawing of life-sustaining therapy in older adults (>/= 80 years) admitted to the intensive care unit. Intensive Care Med. ed. 2018;44:1027–38. 29774388 10.1007/s00134-018-5196-7

[18] Allison BJ, Kaandorp JJ, Kane AD, Camm EJ, Lusby C, Cross CM, et al. Divergence of mechanistic pathways mediating cardiovascular aging and developmental programming of cardiovascular disease. FASEB J. 2016;30:1968–75. 26932929 10.1096/fj.201500057 PMC5036970

[19] Tikkinen KAO, Leinonen JS, Guyatt GH, Ebrahim S, Järvinen TLN. What is a disease? Perspectives of the public, health professionals and legislators. BMJ Open. 2012;2:e001632. 10.1136/bmjopen-2012-001632 PMC3533011 23204142

[20] Smith R. In search of non-disease. BMJ. 2002;324:883–5. 11950739 10.1136/bmj.324.7342.883 PMC1122831

[21] Rabheru K, Byles JE, Kalache A. How old age was withdrawn as a diagnosis from ICD-11. Lancet Healthy Longev. 2022;3:e457–9. 36102756 10.1016/S2666-7568(22)00102-7

[22] Moynihan R. A new deal on disease definition. BMJ. 2011;342:d2548. 21540259 10.1136/bmj.d2548

[23] Conrad P, Barker KK. The social construction of illness: key insights and policy implications. J Health Soc Behav. 2010;51:S67–79. 20943584 10.1177/0022146510383495

[24] Abraham J. The pharmaceutical industry as a political player. Lancet. 2002;360:1498–502. 12433532 10.1016/S0140-6736(02)11477-2

[25] Geriatric Medicine. History of a young specialty. Virtual Mentor. 2014;16:385–9. 24847710 10.1001/virtualmentor.2014.16.05.mhst1-1405

[26] Greenhalgh T, Howick J, Maskrey N, for the Evidence Based Medicine Renaissance Group. Evidence based medicine: a movement in crisis? BMJ. 2014;348:g3725. 24927763 10.1136/bmj.g3725 PMC4056639

[27] Guidet B, de Lange DW, Boumendil A, Leaver S, Watson X, Boulanger C, et al. The contribution of frailty, cognition, activity of daily life and comorbidities on outcome in acutely admitted patients over 80 years in European icus: the VIP2 study. Intensive Care Med. 2020;46:57–69. 2019/12/01. 31784798 10.1007/s00134-019-05853-1 PMC7223711

[28] Schwartz Peter H. Risk and disease. Perspect Biol Med. 2008;51:320–34. 18723938 10.1353/pbm.0.0027

[29] Räikkä J. The social concept of disease. Theor Med Bioeth. 1996;17:353–61. 10.1007/BF00489680 9001128

[30] Maung HH. What’s my age again? Age categories as interactive kinds. HPLS. 2021;43:36. 10.1007/s40656-021-00388-5 PMC7946666 33694016

[31] Sadler JZ, Jotterand F, Lee SC, Inrig S. Can medicalization be good? Situating medicalization within bioethics. Theor Med Bioeth. 2009;30:411–25. 19997778 10.1007/s11017-009-9122-4

[32] Lantz PM, Lichtenstein RL, Pollack HA. Health policy approaches to population health: the limits of medicalization. Health Aff. 2007;26:1253–7. 10.1377/hlthaff.26.5.1253 17848434

[33] Illich I. Medical nemesis. Lancet. 1974;303:918–21. 10.1016/s0140-6736(74)90361-4 4133432

[34] Dunstan GR. Hard questions in intensive care. A moralist answers questions put to him at a meeting of the intensive care Society, autumn, 1984. Anaesthesia. 1985;40:479–82. 4014625

[35] Gajic O, Ahmad SR, Wilson ME, Kaufman DA. Outcomes of critical illness: what is meaningful? Curr Opin Crit Care. 2018;24:394–400. 30045089 10.1097/MCC.0000000000000530 PMC7008960

[36] Fried TR, Bradley EH, Towle VR, Allore H. Understanding the treatment preferences of seriously ill patients. N Engl J Med. 2002;346:1061–6. 11932474 10.1056/NEJMsa012528

[37] Wunsch H, Linde-Zwirble WT, Harrison DA, Barnato AE, Rowan KM, Angus DC. Use of intensive care services during terminal hospitalizations in England and the United States. Am J Respir Crit Care Med. 2009;180:875–80. 19713448 10.1164/rccm.200902-0201OC

[38] Clark D. Between hope and acceptance: the medicalisation of dying. BMJ. 2002;324:905–7. 11950744 10.1136/bmj.324.7342.905 PMC1122840

[39] Heath I. Who needs health care—the well or the sick? BMJ. 2005;330:954–6. 15845979 10.1136/bmj.330.7497.954 PMC556345

[40] Wunsch H. Is there a starling curve for intensive care? Chest. 2012;141:1393–9. 22670019 10.1378/chest.11-2819 PMC3367487

[41] Solberg CT, Barra M, Sandman L, Hoffmann B. Severity as a moral qualifier of malady. BMC Med Ethics. 2023;24:25. 37004054 10.1186/s12910-023-00903-2 PMC10064741

[42] Agledahl KM, Førde R, Wifstad Å. Clinical essentialising: a qualitative study of doctors’ medical and moral practice. Med Health Care Philos. 2010;13:107–13. 20336384 10.1007/s11019-009-9193-z PMC2848348

[43] Reznek L. The Nature of Disease. 1st ed. London: Routledge; 2022. Available from: https://www.taylorfrancis.com/books/9781003283706.

[44] Hofmann B. Expanding disease and undermining the ethos of medicine. Eur J Epidemiol. 2019;34:613–9. 30796581 10.1007/s10654-019-00496-4

